# Obituary

**Published:** 1886-04

**Authors:** 


### Obituary.

Died at his home in New York, of cerebral apoplexy, Dr. Austin Flint, Sr.

Prof. Flint has for many years, and up to the time of his death, occupied the Chair of Practice of Medicine in Bellevue Hospital Medical College.

Dr. Flint was in many respects one of the foremost men of his profession in this country. He was an extensive writer. From his hand have come some of the best medical text-books, and his productions in the periodical literature have always elicited profound attention. No man in the United States was more widely or more favorably known to the medical profession of the world than Dr. Flint. He was President elect of the International Medical Congress to be held in Washington City in 1887. In this matter, as well as in many other directions, the Dr.'s death will be a very great loss.

